# P-870. Daptomycin-Susceptible vs. Daptomycin-Non-susceptible *Enterococcus* Bloodstream Infections: Rates and Predictors of Treatment Failure and Predictors of Daptomycin Resistance

**DOI:** 10.1093/ofid/ofae631.1061

**Published:** 2025-01-29

**Authors:** Kelsey Olion, Ryan P Moenster, Ashleigh Wallace-Lacey, Blake Remensnyder, Travis W Linneman

**Affiliations:** VA St. Louis HCS, St. Louis, Missouri; UHSP/VA St. Louis HCS, St. Louis, Missouri; VISN 15/VA HCS, St. Louis, Missouri; VA St. Louis HCS, St. Louis, Missouri; VA St. Louis HCS, St. Louis, Missouri

## Abstract

**Background:**

Daptomycin is a well-tolerated option to treat resistant enterococci; however, updated breakpoints from the Clinical Laboratory and Standards Institute (CLSI) have led to increasing numbers of ‘susceptible, dose-dependent’ (SDD) or daptomycin non-susceptible (DNS) isolates.

**Methods:**

This study was to determine if isolation of daptomycin-sensitive enterococcal (DSE) or daptomycin-non-susceptible enterococcal (DNSE) isolates from the blood impacts patient outcomes. A retrospective, observational, cohort of patients within the Veteran’s Affairs (VA) Healthcare System from 1 January 2017 to 31 December 2021 with at least 1 blood culture positive for *Enterococcus faecalis* or *E. faecium* was identified. Patients were analyzed in 3 cohorts: 1) all included patients, 2) only patients with minimum inhibitory concentration (MIC) data available, and 3) *E. faecium* isolates and DSE isolates only. The primary outcome of treatment failure was a composite of 30-day mortality and microbiological failure. Two multivariate regressions were conducted - one to establish predictors of treatment failure and one to establish predictors for daptomycin non-susceptibility.

**Results:**

Two hundred four patients were included (90 DSE, 114 DNSE). In cohort 1, 63.3% (57) DSE patients experienced failure vs. 58.8% (67) in the DNSE group (*p* = 0.56). Sixty-nine percent (40/58) of DSE patients failed in cohort 2 vs. 56.4% (35/62) of DNSE patients (*p* = 0.157), and 69% (40/58) of DSE and 64.4% (56/87) of DNSE failed in cohort 3 (*p* = 0.56). Multivariate regression revealed infectious diseases (ID) consultation (OR 0.402; 95% CI, 0.176-0.918; *p* = 0.030) was associated with reduced risk of failure, while infection with vancomycin-resistant enterococcus (OR 2.04; 95% CI 1.028-4.049; *p* = 0.04) and endocarditis/endovascular source of infection (OR 4.33; 95% CI 1.34 – 13.99; *p* = 0.01) were associated with increased risk of failure. Previous beta-lactam and vancomycin exposure were associated with increased risk of DNSE.

**Conclusion:**

No significant difference in failure between patients with DSE and DNSE bacteremias was observed; however, ID consultation was found to be protective, and previous antibiotic exposure was associated with increased DNSE risk.

**Disclosures:**

**All Authors**: No reported disclosures

